# Flares and definition of worsening in inflammatory myopathies: a narrative review of the literature

**DOI:** 10.1093/rheumatology/keag522

**Published:** 2026-09-18

**Authors:** Maria Rosa Pellico, Mert Öztaş, Latika Gupta

**Affiliations:** Clinical Rheumatology Unit, ASST Gaetano Pini–CTO Institute, Milan, Italy; Department of Clinical Sciences and Community Health, Research Center for Adult and Pediatric Rheumatic Diseases, University of Milan, Milan, Italy; Department of Rheumatology, Acıbadem University School of Medicine, Acıbadem Atakent Hospital, Istanbul, Turkey; Department of Rheumatology, Royal Wolverhampton NHS Trust, Wolverhampton, United Kingdom; The Francis Crick Institute, London, United Kingdom; Department of Inflammation and Ageing, School of Infection, Inflammation and Immunology, University of Birmingham, Birmingham, United Kingdom

**Keywords:** myositis, DM, flare, worsening, outcome measures relapse, disease activity, inflammatory myopathies

## Abstract

Idiopathic inflammatory myopathies (IIMs) follow a chronic, polycyclic course in a substantial proportion of patients. Unlike remission, which has recently been standardized, the definition of flare (or worsening) remains highly heterogeneous, creating a divide between clinical trials and real-world practice. Validated core set definitions of worsening exist for trials but were derived by consensus on paper patient profiles and are criticized for inadequate content validity; observational studies instead rely on simplified markers such as enzyme elevation or therapeutic escalation. This review summarizes existing definitions, contrasting the International Myositis Assessment and Clinical Studies Group and Paediatric Rheumatology International Trials Organisation statistical criteria with the variable definitions used in longitudinal cohorts. We examine reported flare rates and predictors, the discordance between global and organ-specific (skin, muscle, lung) worsening, and the lessons offered by flare definitions in other rheumatic and inflammatory diseases. We then propose a multi-domain, treatment-anchored framework intended to bridge statistical rigour and clinical utility.

*Rheumatology* key messages

- There is no standardized definition of flare in idiopathic inflammatory myopathies.
- Current flare definitions vary substantially across clinical trials and observational studies.
- Future consensus should integrate organ-specific activity, patient-reported outcomes and treatment decisions.

## Introduction

Idiopathic inflammatory myopathies (IIMs) are a heterogeneous group of systemic autoimmune diseases including DM, PM, IBM, immune-mediated necrotizing myopathy (IMNM), JDM and antisynthetase syndrome (ASyS) [1]. While induction of remission is the primary therapeutic goal, the natural history of IIM is frequently polycyclic, with periods of inactivity interrupted by distinct episodes of worsening or flare [2].

Accurately defining flare is critical for measuring the durability of response in clinical trials and for guiding immunosuppressive tapering in practice. Broadly, flare denotes the re-emergence of measurable disease activity after a period of stability or improvement; relapse and recurrence are often used interchangeably [2, 3]. International consortia, including the International Myositis Assessment and Clinical Studies Group (IMACS) and the Paediatric Rheumatology International Trials Organisation (PRINTO), have developed standardized core set measures (CSMs) and statistical definitions of worsening [4, 5]. These typically require worsening of ≥30% in at least three of six CSMs, or significant deterioration in physician global activity (PGA) or muscle strength. Although they are the reference standard for trials, they are cumbersome to calculate in routine care.

The difficulty of establishing a universal definition stems from disease heterogeneity. Large real-world cohorts such as the EuroMyositis Registry and the Johns Hopkins Myositis Cohort show that muscle involvement ranges from absent to universal across subsets: ∼20% of patients with DM have clinically amyopathic disease (CADM) with skin manifestations only, muscle involvement in ASyS varies from >90% in anti-Jo-1 disease to 40–50% in non-Jo-1 (often lung-dominant) subtypes, and IMNM and IBM are characterized by near-universal weakness [6, 7]. Pooling such divergent subsets to derive a single holistic criterion risks a definition that underdetects organ-specific worsening in some patients (e.g. pulmonary progression in ASyS) while being excessively sensitive to biochemical fluctuation in others (creatine kinase [CK] elevation in PM or DM). A further challenge is integrating the subjective patient experience with objective measurement, since exacerbations evolve through stages and manifest differently across domains.

In this narrative review, we provide an overview of existing flare and relapse criteria in adult IIM, assess reported recurrence rates and predictors of relapse, and propose a multi-domain framework for defining worsening. Flare in IBM and in juvenile IIM, while important, is beyond the scope of this review; paediatric criteria are used only as methodological comparators.

### Search strategy

We conducted a structured search of MEDLINE and Embase for publications up to January 2026, combining ‘myositis’ with ‘flare’, ‘relapse’, ‘recurrence’, ‘worsening’ and ‘predictors’. After duplicate removal, titles/abstracts and eligible full texts were screened. Studies including adults with IIM and addressing flare/relapse definitions, predictors of worsening or recurrence rates were included. Reference lists were manually screened for additional studies. Global and organ-specific definitions, relapse predictors and recurrence rates were extracted, alongside relevant worsening criteria from other rheumatic diseases. The selection process is summarized in Supplementary Fig. S1. Restriction to English-language reports remains a limitation.

## Disease course, flare terminology and reported rates

IIM trajectories are traditionally classified as monocyclic, polycyclic or chronic continuous, with flare defining the latter two patterns. A polycyclic course, characterized by recurrent activity after documented remission or inactivity, was reported by Ponyi *et al.* in 37.1% of adults with DM and 31.6% of patients with JDM [8], and Bronner *et al.* [2] found that up to 80% of patients followed a polycyclic or chronic continuous course, with sustained monocyclic remission in only a minority. A chronic continuous course denotes persistent activity beyond 2 years despite treatment [9, 10]. The distinction is therapeutically meaningful: worsening in chronic continuous disease represents treatment failure, whereas flare in polycyclic disease signifies reactivation of previously quiescent disease.

Three conceptual definitions recur across cohorts (see Table 1). Clinical relapse denotes a sustained or progressive, measurable decline in strength or function or the recurrence of rash, arthritis or lung disease after stability [3, 11]. Biochemical relapse denotes a rise in muscle enzymes, typically CK to ≥3 times baseline or the upper limit of normal, without alternative explanation; Agarwal *et al.* observed this in 65% of patients, frequently preceding clinical manifestations [11].

**Table 1:** Flare and definition of worsening for idiopathic inflammatory myopathies in observational studies.

First author, year	Cohort (IIM subgroups, number of patients)	Characteristics (age (years), female rate)	Clinical component (symptoms)	Biochemical/other objective component	Flare rate & timing (months)	Identified predictors
Phillips [3], 1998	PM/DM/OM, 50	–	Reduction in muscle strength or reactivation of skin changes	≥3× baseline CK elevation	60%, –	–
Marie [15], 2001	PM/DM, 77	52, 57%	Recurrence of muscle and/or dermatologic manifestations	CK elevation	61%, –	–
Phillips [22], 2002	PM/DM/OM, 53	–	Reduction in muscle strength or reactivation of skin changes	≥3× baseline CK elevation	77%, –	–
Agarwal [11], 2006	PM/DM/OM/CAM, 53	48.8, 71.2%	–	≥3× baseline CK elevation	65%, 17.5 ± 14.4	Older age of diagnosis
Marie [38], 2012	ASyS, 89	54.2, 64%	Reduction in muscle strength or reactivation of skin changes	CK elevation	63.9%, –	–
Taborda [83], 2014	PM/DM/OM/JDM, 90	38.5, 71.4%	–	–	31.1%, –	–
Vuong [12], 2016	DM, 34	46, 71%	Recurrence of muscle or cutaneous manifestations	–	33%, 15 ± 26	Dysphonia, severe skin involvement
Tatebe [14], 2018	PM/DM/CADM, 89	58, 68%	Exacerbation of muscle weakness, ILD, skin rash, arthritis	CK elevation	40%, 11.4 ± 10.6	Anti-SS-A/Ro-positivity
Titou [13], 2020	DM, 33	41.8, 66%	Reoccurrence of muscular or skin manifestations	–	63.6%, 12 ± 14.4	Presence of skin ulceration
Naveen [16], 2021	OM/DM/PM/ASyS/CAM, 214	39 ,81.2%	New or worsening muscle weakness, rash, arthritis or lung disease	CK elevation, worsening lung imaging or pulmonary function tests	49.7%, –	–
Kishaba [58], 2024	DM/PM, 74	57.2, 71.6%	Recurrence of PM/DM symptoms	Increased opacity in chest HRCT	48.6%, –	Serum LDH, GGO
Choi [55], 2024	DM/PM, 105	51.9, 66.6%	Recurrence of muscle and/or dermatologic manifestations	–	61.9, –	ANA titer
Hasegewa [84], 2025	ASyS, 51	60, 68.6%	Recurrence of muscle and/or respiratory manifestations	Continuous CK elevation, decline of pulmonary functions or extending ILD on HRCT or chest X-ray	58%, 36	The presence of anti-EJ/Jo-1 antibody and Gottron’s sign
Hou [17], 2025	DM, 140	43.9, 69.3%	New or worsening muscle weakness, skin lesions, arthritis or lung disease	–	20.7, –	Anti-Mi-2 positivity
Choma [85], 2025	ASyS, 48	44.3, 81.2%	Recurrence of muscle weakness	≥2× baseline CK elevation	41.6%, –	Younger age of diagnosis
Tsuji [82], 2025	DM, PM, IMNM, JDM, IBM, UM patients achieved GCs free remission, *n* = 159	52.5, 62%	≥%30 reduction of MMT8, ≥20-30 increase in PtGA, PGA or extramuscular disease VAS, increase in the HAQ score of ≥0.22–0.25	≥30% CK elevation	76%, over 144	Anti-Jo-1 positivity, baseline cardiac involvement

Abbreviations: ASyS, antisynthetase syndrome; CADM, clinically amyopathic DM; CAM, cancer associated myositis; CK, creatinine kinase; GC, glucocorticoids; GGO, ground-glass opacity; HAQ, health assessment questionnaire; HRCT, high resonance CT; ILD, interstitial lung disease; IMACS, international myositis assessment and clinical studies group; IMNM, immune-mediated necrotizing myopathy juvenile dermatomyositis, LDH, lactate dehydrogenase; MMT8, manual muscle testing-8; OM, overlap myositis; PGA, physician global assessment; PtGA, patient global assessment; UM, unclassified myositis; VAS, visual analogue scale.

Therapeutic flare or treatment failure is defined by the need to escalate immunosuppression or to increase corticosteroids, commonly by ≥50% [12–14]. Marie *et al.* [15] added a temporal anchor, separating short-term relapse occurring during immunosuppressive tapering (47% of patients) from long-term relapse after complete discontinuation (12%), thereby distinguishing drug dependency from true recurrence after drug-free remission. Relapses cluster within the first 2 years of treatment and specifically around tapering [11], and the first post-remission year carries the highest risk [8].

These conceptual differences drive the wide range of reported flare rates, ∼30–80%, which vary further with subset and follow-up duration: rates are consistently highest in PM, DM and SSc-overlap myositis, and lowest in malignancy-associated disease [3, 11, 16]. Cross-cohort comparison is therefore of limited value; definitions and rates are summarized in Table 1.

## Definitions of worsening in randomized controlled trials

Randomized controlled trials have relied on standardized response criteria rather than explicit flare definitions (see Table 2). These are complex, statistically anchored and designed to quantify improvement rather than clinically meaningful relapse; even methodologically robust trials therefore leave unresolved how flare should be defined in IIM.

**Table 2:** Disease activity criteria for idiopathic inflammatory myopathies in clinical trials.

First author, year	Agent	Phase	Cohort (IIM subgroups, number of patients, *n*)	Eligibility
Muscle study group [86], 2011	Etanercept	Phase II	DM, 16	Active DM defined by (a) symmetric proximal weakness, (b) characteristic rash and (c) laboratory evidence of active DM with an elevated serum CK, electromyography demonstrating myopathic features, abnormal skeletal muscle MRI or a muscle biopsy demonstrating perifascicular atrophy and perivascular inflammation.
Oddis [87], 2013	Rituximab	Phase II/III	DM, PM, JDM, 200	Refractory IIM with muscle weakness (MMT < 125/150) plus ≥2 abnormal IMACS core set measures (≥3 for JDM), on stable glucocorticoid ± IS therapy
Tjärnlund [88], 2018	Abatacept	Phase IIb	DM, PM, 20	Adult DM/PM with persisting active disease despite glucocorticoids + ≥1 IS drugs ≥3 months. Active disease was defined as persisting or worsening muscle weakness (MMT-8 bilaterally <150) or low endurance measured by Functional Index for myositis (FI-2) < 20% of upper value, together with at least one other sign of active disease: elevated CK, inflammation in a recent muscle biopsy (<1 month) or on MRI findings consistent with inflammation, or active extra muscular disease
Schiffenbauer [89], 2018	Infliximab	Phase II	DM, PM, 12	Active DM/PM with ≥4 weeks of clinical muscle weakness, on stable prednisone (≤0.5 mg/kg/day) and background IS. Active disease was defined MMT scores between 80 and 120, and active myositis (evidenced by weakness, elevated CK, LDH, aldolase or MRI findings consistent with inflammation
Aggarwal [90], 2022	IVIG	Phase III	DM, 95	Adult DM fulfilling Bohan and Peter criteria and had received previous treatment with a glucocorticoid or other IS drug and had had either no response or an adverse event
Oddis [91], 2022	Tocilizumab	Phase IIb	DM, PM, 36	Adult DM/PM fulfilling Bohan and Peter criteria. Enrolled subjects had ‘active’ disease defined by at least three of six IMACS core set measures. Refractory disease was defined as having failed (or demonstrating intolerance to) an adequate course of glucocorticoids or having failed glucocorticoids and at least one other IS or immunomodulatory agent
Marder [92], 2024	Belimumab	Phase IIb	DM, PM, 17	Adult dermatomyositis or PM with active disease fulfilling Bohan and Peter criteria, on stable glucocorticoids ± conventional immunosuppressants; active myositis defined by IMACS core set (MMT-8 < 125/150 plus ≥2 abnormal domains. Refractory IIM was defined as chronic active IIM with a history of inadequate response or intolerance to 3 months of glucocorticoids and/or at least a history of inadequate response or intolerance to 3 months of one IS agent

Abbreviations: GC, glucocorticoids; HAQ, health assessment questionnaire; IIM, idiopathic inflammatory myopathies; IMACS, international myositis assessment and clinical studies group; IS, immunosuppressants; MMT-8, manual muscle testing-8; PhGA, physician global assessment; PtGA, patient global assessment; ULN, upper limit of normal; VAS, visual analogue scale.

## Organ-specific worsening

Global definitions of worsening rest on composite assessment of muscle and extra-muscular activity, for which therapeutic escalation frequently serves as a proxy in observational cohorts [12–14], sometimes combined with features such as dysphagia or dysphonia and with serum enzymes or inflammatory markers [16, 17]. Because manifestations differ so markedly between patients, global definitions may lack sensitivity to isolated organ deterioration. The Myositis Intention to Treat Activity Index (MITAX) provides structured criteria for worsening within domains (e.g. a shift from category C to B or A), but the observational literature remains variable, with most studies relying on composite ‘clinical recurrence’.

## Skin

Early definitions were global or muscle-centred; Phillips *et al.* [3] first framed skin relapse as the appearance or reactivation of skin changes, distinct from myositic relapse. More recent cohorts have operationalized cutaneous recurrence therapeutically, as the return of skin manifestations requiring a ≥50% increase in corticosteroids or the addition of an immunosuppressant [12, 13].

A cutaneous flare is better conceptualized as a quantifiable increase in inflammatory skin activity, distinct from irreversible damage such as poikiloderma, atrophy or calcinosis [9]. The Cutaneous Dermatomyositis Disease Area and Severity Index (CDASI) was the first robust skin-specific instrument validated to separate activity from damage, allowing flare to be operationalized as a measurable change in score [10]. Building on this, a ≥8-point increase in CDASI activity score has been preliminarily proposed as a cutaneous flare, anchored to a ≥3-point increase in physician global assessment and to meaningful worsening in patient-reported quality of life [18]. The approach is methodologically notable for its anchor-based derivation and triangulation across validated instruments, but its minimal clinically important difference (MCID) was derived from improvement rather than worsening data, is vulnerable to scorer bias and may be obscured by variable follow-up intervals that conflate acute flare with chronic progression.

Cutaneous flares appear to represent interferon-driven, phenotype-specific surges in skin activity, most strongly in anti-MDA5, TIF1γ, SAE and NXP2 disease [19]; candidate biomarkers including CXCL9, CXCL10 and IL-18 correlate with skin activity and fluctuate with treatment [19, 20]. Severe skin involvement at onset (CDASI >20) and ulceration at presentation are the principal clinical predictors of subsequent flare [12, 13]. Ultraviolet exposure frequently precipitates skin-first relapse, with summer clustering; infections, vaccinations, rapid glucocorticoid tapering, antimalarial non-response and immunostimulatory supplements have also been implicated [21].

## Muscle

Muscle flare definitions have oscillated between clinical weakness and biochemical elevation. Phillips *et al.* defined clinical relapse as a sustained or progressive measurable reduction in upper and lower limb strength with functional deterioration [22], which Coppo *et al.* [23] expanded to include electromyographic changes compatible with active myositis. Agarwal *et al.* by contrast, applied a strict biochemical definition of sustained CK elevation above three times the upper limit of normal or baseline [11]. The two constructs are discordant: in that cohort biochemical relapse occurred in 65% of patients overall but in 89% of those with PM, compared with 40% in DM and 33% in malignancy-associated myositis, indicating that enzyme elevation may precede, or occur independently of, functional decline. A similar clinicobiochemical pattern was reported in anti-HMGCR immune-mediated necrotizing myopathy: in a six-patient case series, rising CK closely paralleled recurrent weakness, with five of six patients relapsing during attempts to taper or withdraw corticosteroids and improving after re-escalation of immunotherapy [24].

## Interstitial lung disease

Worsening in IIM-associated interstitial lung disease (ILD) is judged from a combination of symptoms, pulmonary function and imaging, but three distinct constructs are conflated in the literature. Relapse is defined in event-based PM/DM-ILD and mixed IIM-ILD cohorts as new or worsening respiratory symptoms, deterioration in pulmonary function tests or radiological progression requiring treatment modification [25–27]. Progressive fibrosing ILD is captured by applying the 2022 ATS/ERS/JRS/ALAT criteria, focusing on sustained forced vital capacity (FVC) decline, symptom worsening and HRCT progression of fibrosis over 1–2 years [28–31]; this describes chronic fibrotic remodelling despite immunosuppression rather than a discrete inflammatory event. Acute exacerbation, defined analogously to acute exacerbation of idiopathic pulmonary fibrosis as respiratory deterioration over days to weeks with new bilateral ground-glass opacification or consolidation unexplained by infection, heart failure or embolism [32, 33] and rapidly progressive ILD (RP-ILD) are less frequent than relapse or chronic progression (∼10% of IIM) but carry the highest short-term mortality [33, 34], particularly RP-ILD in anti-MDA5-positive DM [35, 36]. Notably, the representation of pulmonary activity within the CSMs, as part of a single extra-muscular global assessment, is limited and highly subjective. In a small anti-EJ antibody-related ILD cohort, deterioration was defined by a >10% fall in FVC or >15% fall in DLCO together with imaging assessment, while relapse required deterioration prompting reinforcement of ILD therapy; five of 12 patients relapsed during a median 74-month follow-up, four while receiving corticosteroid monotherapy [37].

One-quarter to one-half of patients with IIM-ILD responding to initial therapy relapse over subsequent years despite ongoing immunosuppression [26, 38, 39], and a progressive fibrosing phenotype emerges in 30–40% [28, 29]. Serotype is the primary stratifier: anti-MDA5 predicts early RP-ILD and early mortality rather than a relapsing course [35, 36, 40], non-Jo-1 anti-aminoacyl-tRNA synthetase antibodies are associated with chronic, often relapsing ILD [27, 39–41], and overlap antibodies (PM/Scl, Ku, U1-RNP) with severe, ILD-predominant phenotypes prone to chronic progression [28, 30, 31, 42]. Serum lung injury markers (KL-6, SP-D) and immune cell phenotypes such as natural killer and B cell depletion may complement serology, pulmonary function and HRCT in risk models [29, 31]. Structural progression on HRCT correlates with inexorable FVC decline, poor response to immunosuppression and substantially worse survival [28, 30, 31, 43]. Adequacy of immunosuppression is a major modifiable determinant of recurrence: lower initial glucocorticoid dose was independently associated with HRCT deterioration in ASyS-ILD [31], and 26.1% of recurrences in a Chinese IIM-ILD cohort were attributed to drug discontinuation [26].

## Subtype-specific and overlap phenotypes

Manifestations differ so substantially across clinico-serological subsets that a single threshold is unlikely to serve all. IMNM presents with profound, rapidly progressive proximal weakness and CK often above 10 times the upper limit of normal, with myofibre necrosis and minimal inflammatory infiltrate, in contrast to the perivascular and perifascicular inflammation of DM. Recent longitudinal data further suggest that relapse propensity within IMNM is heterogeneous and cannot be adequately captured by serological subtype alone. In 138 patients with IMNM, integrated clinical–serological–pathological clustering identified phenotypes with markedly different times to remission and relapse-free survival, whereas stratification by anti-SRP, anti-HMGCR or seronegative status was less discriminative [44].

CADM and hypomyopathic DM show minimal muscle involvement despite active cutaneous disease, rendering muscle-based definitions inadequate, while flares in ASyS may manifest predominantly as ILD progression, inflammatory arthritis or mechanic’s hands [1]. Fibrotic PM is characterized by progressive atrophy and replacement fibrosis with minimal inflammation, so inflammatory markers may remain normal despite functional decline [45]. Clinico-serological classification may therefore be more relevant than umbrella diagnostic categories when defining flare.

Overlap syndromes expose a deeper conceptual problem. In scleromyositis the scleroderma component progresses fibrotically without discrete flares, while the myositis component relapses and remits [46]. Inflammation, necrosis and fibrosis occur simultaneously in the same tissue, so clinical worsening alone cannot distinguish a treatable inflammatory flare from irreversible scarring, in which immunosuppression may be ineffective or harmful. The field has no consensus on whether overlap syndromes should adopt flare-based definitions (as in myositis), progression-based definitions (as in scleroderma) or a dual framework. We favour the last: worsening should be scored separately for inflammatory activity and fibrotic progression, so that irreversible fibrosis is not mistaken for reversible inflammation.

## Patient-reported outcomes and functional measures

Patient-reported outcomes (PROs) capture symptoms that often precede objective signs. Christopher-Stine *et al.* found that >70% of patients with DM or PM reported more than one flare annually, most commonly muscle weakness (83%), fatigue (78%) and pain [47], and higher patient-reported flare frequency correlated with greater disability, reduced work productivity and increased emergency and urgent care utilization [48]. Patient-reported flare frequency therefore functions as a marker of deteriorating physical function and escalating healthcare need. Exploratory smartphone-based approaches to early flare identification are promising but require validation [49].

Functional deterioration is a key component of relapse definitions. PROMIS Physical Function-20 showed strong test–retest reliability, convergent validity and responsiveness in 50 adults with IIM, and outperformed the HAQ-DI, which exhibited ceiling effects, and the SF-36 PF10 [50]. Ongoing OMERACT core set work provides a framework to enrich future flare definitions with these instruments [51].

## Composite measures: response vs worsening

The total improvement score (TIS), derived from the ACR-EULAR myositis response criteria, integrates change across the six IMACS CSMs with greater weight given to muscle strength and physician global assessment, discriminating minimal, moderate and major response (TIS ≥20, ≥40 and ≥60, respectively) [52]. Saygin *et al.* [53] validated these criteria in 412 adults with DM or PM: 95% of responders showed muscle-related improvement, and minimal responders improved in a median of three CSMs vs six for major responders, although derivation from three trials and consensus paper profiles limits real-world generalizability. An anchor-based study in 105 patients, using an SF-36 physical component summary improvement of ≥7.2 points as the anchor, suggested an MCID of ∼24 TIS points rather than the consensus 20, with corresponding thresholds for muscle strength (∼4 points), function (0.65) and patient and physician global assessments (1.84 and 1.1 cm) [54]; the contrast with real-world MyoNET registry data underscores the differing performance of TIS in trial and routine settings [55].

Worsening has been far less uniformly defined. The PRINTO definition conceptualizes deterioration as a clinically meaningful decline driven by physician judgement rather than isolated variable fluctuation, requiring ≥30% worsening in at least three CSMs with no more than one improving by >20% [56]. This aligns with intention-to-treat instruments such as MITAX, reinforcing that objective change is best interpreted in the context of therapeutic decision-making [56].

## The myositis disease activity assessment tool and treatment-anchored activity assessment

The Myositis Disease Activity Assessment Tool offers an organ-specific alternative to global assessment through dual scoring: MITAX, which categorizes activity by therapeutic intent, and the Myositis Disease Activity Assessment Visual Analogue Scales, which quantify severity continuously, across seven domains (constitutional, cutaneous, skeletal, gastrointestinal, pulmonary, cardiovascular, muscle) [57]. MITAX grades each domain from A to E, delineating active disease, low disease activity and remission according to the physician’s intention to treat [57]. Because the grading is anchored to what the clinician would do rather than to a percentage change in a continuous variable, it is directly translatable to a definition of flare: a major flare corresponds to category A activity, requiring high-dose corticosteroids or a new disease-modifying agent, and a moderate flare to category B, requiring more modest escalation. This treatment-anchored logic underpins the framework proposed below.

## Biomarkers and imaging

Biochemical relapse is most often defined by a CK rise to ≥3 times baseline or the upper limit of normal [3, 11, 22], with elevated lactate dehydrogenase and aldolase also associated with relapse [58]. In antibody-defined IMNM, longitudinal serological markers may provide additional information on disease activity: anti-HMGCR titres correlated with contemporaneous and troponin T levels in a recent long-term cohort. However, whether antibody titres can prospectively predict flare or safely guide treatment withdrawal remains to be established [59]. Anti-Ro52 positivity independently predicts relapse, whereas anti-U1-RNP antibodies are associated with a more favorable course [14, 60]. High-titre ANAs and elevated ferritin are also linked to relapse and poorer outcomes [55]. In anti-MDA5 DM, ferritin predicts rapidly progressive ILD and mortality and is used to monitor ILD activity [61–69].

## Lessons from flare definitions in other diseases

Worsening criteria in other rheumatic and inflammatory diseases have matured from subjective gestalt into data-driven indices (see Table 3). Four lessons are directly transferable to IIM.

**Table 3:** Flare and definition of worsening in non-IIM autoimmune rheumatic diseases.

Diseases	Definitions	Relapse/worsening criteria
Inflammatory arthritis	DAS28	Formula based on three parameters: TJC and SJC (28- joint assessment), PtGA (on a 0–10 scale): flare an increase in DAS28 of >1.2 between visits, or an increase of >0.6 if the current DAS28 is ≥3.2 [81]
RA		
	SDAI	A sum of five outcome parameters: TJC and SJC (28-joint assessment), PtGA and PhGA (on a 0–10 scale), and CRP: flare an absolute increase in SDAI is ≥4.7 [82]
	CDAI	SDAI modification, eliminating CRP; flare an absolute increase in SDAI is ≥4.5^82^
AxSPA	ASDAS	Based on five items: back pain, peripheral joint pain, duration of morning stiffness, PtGA and CRP (ASDAS-CRP) or ESR (ASDAS-ESR); flare an increase in the ASDAS score of ≥0.9 points [83]
PsA	Patient reported flare	Patient confirmation of symptoms worse than usual [84]
Gout	Patient-reported flare	Presence of at least three of four criteria: (1) patient-reported flare, (2) patient-reported pain at rest >3 (0–10 scale), (3) patient-reported swollen joint, (4) patient-reported warm joint [85]
Connective tissue disorders		
SLE	SELENA-SLEDAI flare index (SFI)	The SFI consists of five components: (1) increase in SLEDAI score, (2) new or worsening clinical manifestations, (3) rise in PGA, (4) intensification of prednisone or immunosuppressive therapy and (5) hospitalization due to lupus [86]
		Mild/moderate flare: change in SLEDAI score ≥3, new/worse discoid rash or increase in prednisone but not >0.5 mg/kg/day
		Severe flare: change in SLEDAI score >12, new/worse CNS involvement, vasculitis, nephritis or need for prednisone >0.5 mg/kg/day or new immunosuppressive
dcSSc	MRSS	The MRSS is measure of skin thickness in dcSSc, assessed across 17 body areas scored from 0 to 3 for a total score ranging from 0 to 51 Worsening: an increase in MRSS >5 points and ≥25% from baseline to second visit [87]
CTD-ILD	Clinically meaningful progression	Progression: ≥10% relative decline in FVC or ≥5–<10% relative decline in FVC% and ≥15% relative decline in DLCO [88]
Vasculitis		
AAV	BVAS	Summation of nine variables: constitutional, skin, mucous membranes/eyes, ENT, chest, cardiovascular, gastrointestinal, renal and nervous system Minor flare: recurrence of ≥1 item on the (BVAS) > 0 not threatening a vital organ [89] Major flare: recurrence of disease activity (BVAS >0) that is organ- or life-threatening (e.g. pulmonary hemorrhage, glomerulonephritis) [89]
PMR	PMR-AS	Summation of five variables: patient’s pain assessment, PGA, morning stiffness duration, CRP level and the ability to elevate the upper limbs. Flare: a PMR-AS score >9.35 or an increase PMR-AS score >6.6 between visits [90]
VEXAS	Disease flare	Active inflammatory manifestation(s) of VEXAS syndrome requiring escalation in glucocorticoid therapy. Three flare categories were established: (i) recurrence of *a prior* documented VEXAS manifestation; (ii) development of a new VEXAS-defining inflammatory manifestation or (iii) emergence of a new inflammatory manifestation not meeting criteria for A or B [91]

Abbreviations: AAV, ANCA-associated vasculitis; ASDAS, ankylosing spondylitis disease activity score; AxSpA, axial SpA; BVAS, Birmingham vasculitis activity score; CDAI, clinical disease activity index; CTD-ILD, connective tissue disease-associated interstitial lung diseases; DAS28, disease activity score-28; dcSSc, diffuse cutaneous SSc; DLCO, diffusion capacity in carbon monoxide; FVC, forced vital capacity; MRSS, modified Rodnan skin score; PGA, physician global assessment; PhGA, physician global assessment; PMR-AS, polymyalgia rheumatica activity score; PtGA, patient global assessment; SDAI, simplified disease activity index; SELENA-SLEDAI, Safety of Estrogens in Lupus Erythematosus National Assessment—SLE Disease Activity Index; SFI, SELENA–SLEDAI Flare Index; SJC, swollen joint count; TJC, tender joint count; VEXAS, vacuoles, E1 enzyme, X-linked, autoinflammatory, somatic.

Flare should be quantified as change, not activity. In RA and axial SpA (AxSpA), flare is defined mathematically as an increase in a continuous composite score, providing a precise delta that distinguishes true reactivation from measurement error: DAS28, SDAI or CDAI in RA [70, 71], and an ASDAS increase of ≥0.9 points for ‘clinically important worsening’ in AxSpA [72]. Such thresholds permit direct cross-trial comparison of durability of response, a metric currently impossible to standardize in myositis. Critically, flare must capture both change and severity: not simply how active the disease is, but how much worse it has become relative to the patient’s own baseline [73].

Flare should be anchored to a treatment decision. The SELENA-SLEDAI Flare Index stratifies flares as mild/moderate or severe, and defines severity not by symptoms alone but by the necessity to escalate prednisone above 0.5 mg/kg/day or to start new immunosuppression [73]. This action-based approach filters out minor, non-specific fluctuation and ensures that only clinically meaningful deterioration triggers a flare end point. The 2024 VEXAS consensus adopted the same logic, centring its definition on glucocorticoid escalation [74]. Definitions must also state explicitly when a flare ends, conventionally at return to the pre-flare level rather than to remission [73].

Organ-specific and tiered definitions accommodate heterogeneity. SLE, the closest multisystem analogue to IIM, defines flare separately by organ system [75] and layers global (SLEDAI), organ-specific (BILAG, with A–E grading) and treatment-based criteria [76], a strategy directly applicable to separating muscle, skin, lung and joint worsening in IIM rather than treating all deterioration as equivalent. The VEXAS consensus similarly used three hierarchical categories, including a deliberate catch-all for atypical inflammatory presentations, on the reasoning that rigidity would exclude legitimate flares in a heterogeneous rare disease, and retained adjudication committees for ambiguous cases [74].

The patient’s perception must be represented but cannot stand alone. The 2012 gout consensus explicitly includes patient-defined flare as a core criterion, recognizing that patients detect prodromal pain or fatigue before objective signs appear [77]. In Sjögren’s syndrome, poor correlation between symptoms and objective findings led to a PRO (ESSPRI, MCID 1 point or 15%) becoming the primary activity measure [78], demonstrating that PRO-based scores can function as standalone measures where objective biomarkers are lacking, a situation approximated in myositis, where enzymes frequently dissociate from symptoms. The hidradenitis suppurativa consensus went further, rejecting the trial-standard ‘≥25% increase in lesion count’ for unestablished validity, low sensitivity and poor interrater reliability, and settling after seven Delphi rounds on a deliberately unquantified definition: new or substantial worsening of clinical signs or symptoms [79]. Counterbalancing this, patients with inflammatory bowel disease perceive flare almost entirely through symptoms, with rare reference to inflammation or tissue damage [80], so patient-defined flares may not correspond to treatment-responsive inflammation, a tension acutely relevant to myositis overlap syndromes, where symptoms may reflect fibrosis rather than activity. IIM definitions in the observational literature nonetheless largely lack any patient-centred component, relying on physician assessment or biochemistry that may be discordant with the patient’s experience.

## Current unmet needs

Published definitions of worsening in adult IIM are limited and highly variable. The validated IMACS and PRINTO criteria, derived by statistical consensus on paper patient profiles, are rarely used outside trials, while observational studies rely on isolated enzyme elevation or physician gestalt. Early definitions were particularly simplistic, resting on weakness and enzymes and failing to account for extra-muscular phenotypes such as ILD or skin-predominant disease.

The original consensus definitions were built on paper patient profiles derived from older natural history data that under-represented rare subsets and now well-recognized phenotypes. Classification has since moved from the Bohan and Peter criteria to the 2017 EULAR/ACR criteria and to antibody-defined subgroups such as ASyS and IMNM, and numerous outcome measures have been validated in the interim [81]. These definitions are best regarded as partially validated tools requiring revision, not settled standards.

The evidence that they underperform is now direct. In a Swedish registry study of 159 patients achieving glucocorticoid-free remission, 76% relapsed over 12 years, with anti-Jo-1 positivity doubling the risk, yet the IMACS worsening criteria captured only half of clinically significant relapses [82]. Four failure modes were identified: short intervals between visits produced small measured changes despite ongoing deterioration; high baseline values prevented percentage-change thresholds being met even when absolute values remained abnormal; the 15% change thresholds for CK and HAQ were too stringent given the low baselines typical of remission (met by only 23% and 50% of patients, respectively) and many relapses manifested through treatment escalation (35%), or were detectable only when change was measured from remission to any subsequent timepoint rather than between consecutive visits (a further 15%). Physician-determined remission captured disease state better than the rigid criteria.

Two structural gaps follow. First, there is a gap between response and flare: clinical practice and trials rely on the ACR-EULAR response criteria, which measure relative improvement from baseline, with no accepted equivalent for loss of that response. ‘Time to flare’ is a critical end point for durability, yet trials defining it by therapeutic escalation cannot be compared with trials defining it biochemically. Second, historical definitions applied uniform weighting across domains, in practice privileging muscle and under-weighting skin and lung [81]. Definitions leaning on CK, which identifies 89% of flares in PM, may miss vasculopathic cutaneous flares or progressive ILD in DM and CADM, in whom enzymes and strength may remain normal despite severe extra-muscular activity, delaying escalation and driving irreversible damage such as scarring or pulmonary fibrosis. Any workable definition must also distinguish true inflammatory relapse from damage, deconditioning, medication effect and, in overlap disease, fibrotic progression.

## A proposed multi-domain framework

Synthesizing the above, we outline a provisional conceptual framework for future definitions of flare in adult IIM that may be defined as a multi-domain, treatment-anchored event with four components.

### Domain-specific triggers

Worsening is assessed independently in muscle, skin, lung, joint, gastrointestinal and constitutional domains, and flare could consider deterioration within individual domains, rather than requiring a threshold number of CSMs to change in parallel. Candidate domain-specific thresholds for future validation may be drawn from existing instruments and observational definitions, including clinically meaningful fall in manual muscle testing with functional loss, with sustained CK elevation ≥3× baseline as a supportive but not sufficient criterion; skin: an increase in CDASI activity score of ≥8 points, or new ulceration, erythroderma or rash with secondary necrosis; lung: a ≥10% relative decline in FVC or DLCO, or new parenchymal abnormality on HRCT; gastrointestinal: new or worsening moderate-to-severe dysphagia; with the MITAX A/B categories used to grade severity across all domains. These thresholds should be regarded as illustrative rather than validated flare criteria.

### Treatment anchoring

Consistent with SELENA-SLEDAI, VEXAS and MITAX, treatment escalation could provide an anchor for flare severity, with a major flare defined when it prompts high-dose glucocorticoid or a new disease-modifying agent (MITAX category A), and a moderate flare when it prompts lesser escalation (category B). Such an approach may help distinguish clinically meaningful deterioration from minor fluctuations while retaining clinical interpretability.

### Patient anchoring

A patient-reported worsening item and a validated functional measure such as PROMIS Physical Function-20 should be recorded at every assessment and reported alongside the physician-defined event, both because patient-perceived flare predicts disability and healthcare use [47, 48] and because concordance between the two is itself informative.

### An activity–damage distinction

In overlap and fibrotic phenotypes, inflammatory activity and fibrotic progression should be scored separately, and progression alone should not be labelled flare. Change should be measured from the last documented remission or low-activity state, not only from the preceding visit, to avoid the interval-dependence that undermines current criteria [82].

Such a framework requires formal Delphi consensus and prospective validation in longitudinal cohorts, with attention to content validity, the principal criticism of existing paper-profile-derived definitions and to responsiveness across serologically defined subsets.

## Conclusion

Flare in IIM is currently defined in as many ways as there are cohorts, and the statistical criteria available for trials are neither used nor usable in practice. Establishing a data-driven definition that weights organ-specific endpoints appropriately, anchors worsening to treatment decisions, incorporates the patient’s experience and distinguishes inflammation from irreversible damage could improve the consistency and clinical relevance of flare assessment across observational studies and clinical trials.

The framework proposed here should therefore be considered hypothesis-generating and will require expert consensus and prospective validation before clinical or research implementation.

## Supplementary Material

keag522_Supplementary_Data

## Data Availability

No new data were generated or analysed in support of this review.

## Notes

*Disclosure statement:* L.G. is an Associate Editor of *Rheumatology.* The other authors have declared no conflicts of interest.
