# Supplementary material for: Flares and definition of worsening in inflammatory myopathies: a narrative review of the literature
Source: Rheumatology (Oxford). 2026 Sep 18;65(10):keag522. doi: 10.1093/rheumatology/keag522 (PMC13630441; doi:10.1093/rheumatology/keag522)
Supplement: Supplementary file 1 [file keag522_supplementary_data.docx]

**Supplementary Figure S1.** Flow diagram of literature identification and study selection
